# How to Survey Citizens’ Compliance with COVID-19 Public Health Measures: Evidence from Three Survey Experiments

**DOI:** 10.1017/XPS.2020.25

**Published:** 2020-07-07

**Authors:** Jean-François Daoust, Richard Nadeau, Ruth Dassonneville, Erick Lachapelle, Éric Bélanger, Justin Savoie, Clifton van der Linden

**Affiliations:** 1 Politics and International Relations, University of Edinburgh, Scotland, UK, Twitter: @jf_daoust; 2 Center for the Study of Democratic Citizenship, Québec, Canada; 3 Department of Political Science, Université de Montréal, Montréal, Québec, Canada, Twitter: @r_dassonneville, @erickUdeM; 4 Department of Political Science, McGill University, Montréal, Québec, Canada; 5 University of Toronto, Toronto, Ontario, Canada, Twitter: @justinsavoie; 6 McMaster University, Hamilton, Ontario, Canada, Twitter: @CliffvdLinder

**Keywords:** COVID-19, public health, self-isolation, compliance, measurement, survey experiment, methodology

## Abstract

The extent to which citizens comply with newly enacted public health measures such as social distancing or lockdowns strongly affects the propagation of the virus and the number of deaths from COVID-19. It is however very difficult to identify non-compliance through survey research because claiming to follow the rules is socially desirable. Using three survey experiments, we examine the efficacy of different ‘face-saving’ questions that aim to reduce social desirability in the measurement of compliance with public health measures. Our treatments soften the social norm of compliance by way of a short preamble in combination with a guilty-free answer choice making it easier for respondents to admit non-compliance. We find that self-reported non-compliance increases by up to +11 percentage points when making use of a face-saving question. Considering the current context and the importance of measuring non-compliance, we argue that researchers around the world should adopt our most efficient face-saving question.

Mass compliance with recently enacted public health measures such as social distancing or lockdowns can have a defining impact on transmission of the novel coronavirus and, by extension, the number of COVID-19-related hospitalisations and deaths.^1^ It is thus fundamental to understand which groups within a population comply with the mitigatory measures set by governments and public health officials, and what motivates citizes to comply with these measures. This challenge has been well understood by the scientific community and several scholars have already examined the question of citizens’ compliance.^2^

However, it is important to carefully consider how best to measure compliance before exploring what determines it. This is especially true given the concern of social desirability, that is, the pressure for someone to claim to have followed a norm. In this research note, we develop three different versions of ‘face-saving’ strategies, which loosen the social norm, to measure compliance with social distancing. While such a strategy has been shown to be useful with regard to such other behaviours as voter turnout, it has not, to date and to our knowledge, been implemented to measure self-reported compliance with COVID-19-related preventive measures. Using three survey experiments, we find an increase in self-reported non-compliance of up to +11 percentage points when citizens receive face-saving treatments. The implication is straightforward: Given that there is no cost of using the face-saving treatment instead of a yes/no question, we stronlgy encourage researchers around the world to include face-saving questions when surveying about compliance with social distancing policies.

## An extraordinary sensitive question

On most topics in life, people know what corresponds to a socially ‘desirable’ and ‘undesirable’ answer. This becomes even clearer in a pandemic where public health explains which behaviours are or not desirable (or even illegal). While behavioural measures of compliance are increasingly relied on (such as COVID-19 tracing applications^3^), survey research remains the most widely used approach to study the attitudinal and motivational correlates of behaviour. For example, it is used to study who makes donations to charities (Clements et al., 2015; Bekkers and Wiepking 2011; Lee and Woodliffe 2010), or closer to the actual context in which frequent and careful hand-washing is crucial for limiting the spread of infection, the extent to which people adequately wash their hands (Contzen et al. 2015).

The risk with using surveys to capture citizens’ attitudes and their behaviour, however, is that survey measures often suffer from social desirability (see Gnambs and Kaspar 2015 for a meta-analysis on the topic). A good example of a question that is hindered by a social desirability bias is voter turnout. When asked whether they would feel guilty if they would not vote, some citizens express a great deal of guilt (Blais and Daoust 2020: 49–50). As a result, there is almost always an overestimation of voter turnout in election surveys. Faced with this problem, Morin-Chassé et al. (2017) developed a survey experiment to test a ‘face-saving’ strategy in election surveys. Specifically, they made use of a short preamble which stated that in each election, some people do not vote. Among the answer choices to the turnout question, they additionally included ‘I usually vote but didn’t this time’ as an option. Morin-Chassé et al. (2017) show that using these face-saving questions provides an effective means to limit overreporting. Their treatment reduces over-reporting of turnout by +4–8 percentage points.

Our work is inspired by the study of Morin-Chassé et al. (2017), from which we learn that the ingredients of an effective face-saving treatment include a short preamble rationalising the non-compliant behaviours and providing a guilty-free answer choice. These are precisely the kinds of treatments that we implemented in three survey experiments for measuring compliance with COVID-19 public health measures.

## Method

Our data come from Canada, where public health measures such as social distancing and lockdowns were taken very seriously and rapidly. Some police services obtained extraordinary power to administer large fines to citizens who were not respecting social distancing. Canadian provinces have considerable autonomy in the management of the crisis; however, for our purposes, these differences are fairly limited, and public health measures were in place in all provinces at the time of our fieldwork.^4^ The data for Study 1 and Study 2 come from Vox Pop Labs, while data for Study 3 were collected by Léger Marketing. Samples from all three studies are nationally representative based on age, sex, region, language and education. Table 1 summarises the dates on field and the number of observations in each study, and we provide details on the selection of the respondents, how their characteristics are similar across studies and weights in the Supplementary Materials. Study 2 and Study 3 were preregistered (link is not shown for the review process).

**Table 1:** Experimental Treatments

	Dates on field	Control group question	Answer choices	Face-saving question	(Guilty-free) answer choices
Study 1 (*n* = 2,607)	April 3^rd^ to 7^th^	Have you done any of the following activities in the last week?	Yes No Unsure	Some people have altered their behaviour since the beginning of the pandemic, while others have continued to pursue various activities. Some may also want to change their behaviour but cannot do so for different reasons Have you done any of the following activities in the last week?	Yes No Unsure
Study 2 (*n* = 2,350)	April 17^th^ to 21^st^	*Idem*	*Idem*	*Idem*	Yes As seldom as possible No Unsure
Study 3 (*n* = 1,006)	April 15^th^ to 21^st^	*Idem*	*Idem*	*Idem*	Yes Only when necessary/occasionaly No I don’t know

In each of the three studies, a random half of the sample was exposed to a face-saving treatment, while the other half served as the control group. The question for the control group was always the same, that is, ‘Have you done any of the following activities in the last week?’, and was followed by different items (displayed in random order).^5^ Turning to the experimental treatments, they all included this short preamble:

Some people have altered their behaviour since the beginning of the pandemic, while others have continued to pursue various activities. Some may also want to change their behaviour but cannot do so for different reasons.

Have you done any of the following activities in the last week?

In Study 1, the treatment was limited to this preamble. In Study 2 and Study 3, we additionally manipulated the answer choices. All respondents in Study 1, and in the control groups of Study 2 and Study 3, could choose between the options ‘yes’, ‘no’ and ‘unsure’. In Study 2, we added a face-saving answer-option that read ‘As seldom as possible’. This answer choice was included as a means of reducing guilt when reporting non-compliance. Study 3 followed a similar design as Study 2, but the guilty-free answer choice in this case was ‘Only when it was necessary/occasionally’. There is thus a gradation in terms of easiness to admit non-compliance from Study 1–3. As indicated in the preregistration, we take any answer other than ‘no’ as an indicator of non-compliance.

## Results

**Figure 1:**
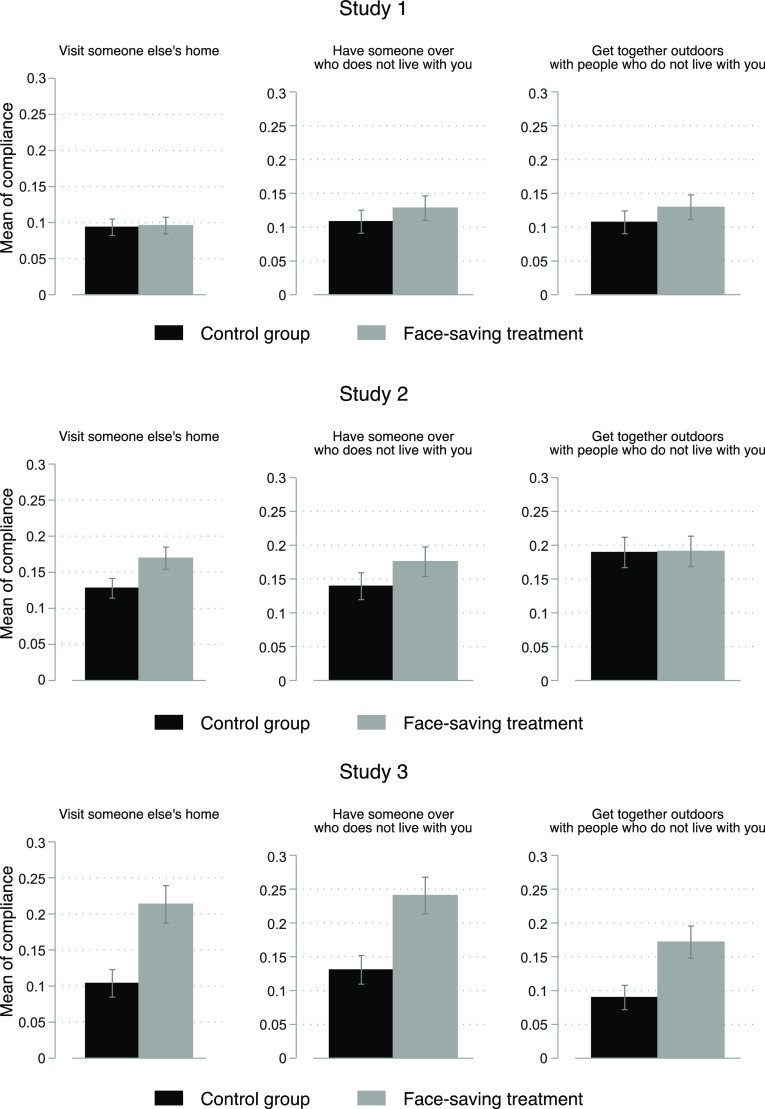
Level of Compliance with COVID-19 Public Health Measures.

*NOTES*: 84% confidence intervals are shown.

Before turning to our main findings, we show in Supplementary Material Table 1 that there is balance between the control and treatment groups regarding age, gender, education, region and left–right ideology.^6^ Hence, we focus on bivariate relationships, which we take as the most appropriate tests (Mutz et al. 2019). Figure 1 displays the mean of compliance for different prohibited activities with 84% confidence intervals.^7^

The first observation is that the means of non-compliance are low. More specifically, on average 11% of the respondents in Study 1 reported visiting someone else’s home, 13% had someone over and 11% met with a group outside. These proportions increase to 14%, 15% and 18%, respectively, in Study 2. The percentages of non-compliance found in Study 3 are similar to Study 2 (16%, 19% and 12%).^8^ The second clear feature is that in all nine cases (three behaviours across three studies) the proportion of non-compliance is greater with the face-saving treatment. Third, these differences vary across studies and the effects are substantially larger in Study 3.

Looking first at Study 1, we find small effects of about +2–3 percentage points. Recall that this study included the face-saving preamble *only* (no guilty-free answer choice) and is thus the weakest treatment. Turning to Study 2, respondents receiving the face-saving treatment were always more numerous in admiting non-compliant in all three items. The proportion of non-compliance increased by about +3 percentage points two out of three items.

In Study 3, we replaced the ‘As seldom as possible’ response option by a potentially greater social desirability inhibiter: ‘Only when necessary/occasionaly’. It allows the respondent to claim that it was morally right to do so (i.e. *necessary*) or that it is only done from time to time, thus approaching a guilty-free answer choice. Supplementary Material Figure 1 shows the distribution of Study 3 responses, breaking down the answer choices and showing that about as many people who admit non-compliance do so using the guilty-free option.

The face-saving strategy appears effective. On average, the effects are of +11 percentage points (seeing someone), +11 percentage points (having someone over) and +8 percentage points (getting together outdoors with people who do not live with you). These effects are important in and of themselves, but they are even more important when we consider the baseline levels of admitted compliance. In fact, the face-saving treatment *doubles* our ability to identify the number of non-compliers.

Our research design applied to our three studies does not allow us to fully disentangle the relative effects of the face-saving prompt (i.e. the preamble) from the guilt-free options, and as a result, we cannot definitively conclude that a guilt-free option would be sufficient to obtain more accurate measures of non-compliance. We can conclude from Study 1 that the preamble alone is not sufficiently strong to reduce social desirability. However, it is not impossible that the guilty-free answer choices are only effective in combination with a face-saving preamble.

Finally, we probe the robustness of our results by including the covariates used for the balance tests as shown in Supplementary Material Figure 2 and by not using the weights (see Supplementary Material Figure 6). We also explore heterogeneity and find that the treatment effects do not substantialy differ by gender, education or ideology (see Supplementary Material Figures 3–5). Out of 27 moderation effects explored, 2 display a differentiated impact (*p* < 0.05) of the treatment: (i) ‘having someone over’ and ideology in Study 1 (Supplementary Material Figure 3) and (ii) ‘having someone over’ and gender in Study 3 (Supplementary Material Figure 5).

## Discussion

We have rarely seen such an urgent need for methodological innovation to improve our understanding of human behaviour and to measure human behaviour well. If we want to be confident in our analyses of a variable such as compliance with social distancing or lockdowns, we must minimise the social desirability bias typically associated with measures of compliance with public health guidelines. We believe that we can do better than what the actual state of research is doing and developed face-saving strategies.

The effects are as predicted in the preregistration of our studies. In all cases, the proportion of non-compliance is greater among those who received the face-saving treatment. Most importantly, the effects were very important in the most effective face-saving treatment (Study 3), allowing to roughly double the number of respondents admitting non-compliance.

Our results contrast with the ones of Larsen et al. (2020) who, using a list experiment of Danish people, found no social desirability bias to admit non-compliance. While we cannot know if the differences with our results are due to sampling differences, or whether the patterns observed in our findings are specific to the Canadian case, we have good reasons to be confident in our results. In particular, our results are systematically in the same direction. Moreover, although our study was conducted in a single country, the Canadian experience with multiculturalism (with more than a quarter of the population speaking French at home) provided an opportunity to show that our results are robust and very similar across these different cultures.^9^ This being the case, replication in other countries such as the Danish case would be useful additions.

Considering the current context and the importance of measuring non-compliance, we argue that researchers and policymakers around the world should adopt the face-saving question. The cost of adding a very short preamble and a guilty-free answer choice is almost *null* and the benefits are clear: Researchers will enhance the validity of their measures, improve their descriptive inferences and be able to identify more non-compliers. In turn, this will enhance causal inferences and produce better analyses when the predicted outcome is compliance vs. non-compliance by getting closer to the true distribution of non-compliance.

## Footnotes

1 We declare no conflict of interest. The data, code, and any additional materials required to replicate all analyses in this article are available at the Journal of Experimental Political Science Dataverse (Daoust et al. 2020) within the Harvard Dataverse Network, at: https://doi.org/10.7910/DVN/7QYFLI.

2 Among others, see Barari et al. (2020) for an in-depth case study of Italy, or Daoust (2020) for a comparative perspective among 27 countries.

3 While useful, the major disadvantage is that it does not include individuals’ opinions, attitudes, etc.

4 For more information, see the data set from Breton and Mohy-Dean (2020) including a detailed timeline for all provinces. All provinces declared the state of emergency between 14^th^ and 22^nd^ March.

5 As indicated in our preregistration report, we focus on the three explicitly prohibited items (displayed in Table 1).

6 Out of 36 *t*-tests, only 5 (in bold) show a *t*-value greater than 1.96.

7 See MacGregor-Fors and Payton (2013) on why the 84% instead of the conventional 95% confidence intervals.

8 While the distributions do not affect our inferential goal, that is, to examine the effect of being exposed to the face-saving question, the lower means in Study 1 cannot be attributed to a different organization collecting the data since Study 2 was also collected by Vox Pop Labs and displays means similar to Study 3, collected by Léger Marketing.

9 In Supplementary Material Figure 8, we show that respondents who filled the surveys in English or French were not significantly different in eight out of nine cases.

## Supplementary material

For supplementary material accompanying this paper visit http://dx.doi.org/10.1017/XPS.2020.25. click here to view supplementary material
