# Supplementary material for: How to Survey Citizens’ Compliance with COVID-19 Public Health Measures: Evidence from Three Survey Experiments
Source: Journal of Experimental Political Science. 2020 Jul 7:1–8. doi: 10.1017/XPS.2020.25 (PMC7438621; doi:10.1017/XPS.2020.25)
Supplement: Supplementary file 1 [file S2052263020000251sup001.docx]

**Supplementary Material**

**Daoust et al. (2020)**

***Journal of Experimental Political Science***

**How to survey citizens’ compliance with COVID-19 public health measures.**

**Evidence from three survey experiments**

**Description of the samples**

Respondents in Study 1 and 2 were drawn from the Vox Pop Labs’ *voluntary, opt-in* web panel that includes 650,000 Canadians. Both samples are part of a 24-wave rolling cross-sectional study (about 2,250 respondents are interviewed in each wave). All 24 waves were drawn simultaneously from the web panel on March 16, with the probability of inclusion in the sample being proportional to Canadian Census data. The data for Study 1 (n=2,607) were collected between April 3^rd^ and April 7^th^ 2020 and the data for Study 2 (n=2,350) were collected between April 17^th^ and April 21^st^ 2020 as part of the fourth and sixth waves (respectively) of the aforementioned rolling cross-sectional study.

The experiment in Study 3 was administered to a Census-balanced, representative sample drawn from the voluntary Leger 360 online platform, which also is *a voluntary, opt-in* web-based panel of over 400,000 Canadian respondents. The data were collected between April 15^th^ and April 21^st^ 2020. Study 2 and 3 were preregistered on OSF. Table SM.1 below shows the means for age, sex, language, region (province) and education, with and without weight that correct for the imbalance between these variables and the latest Canadian census.

Table SM.1: descriptive (weighted) statistics for all studies

|  | Study 1 (n=2,605) | Study 2 (n=2,350) | Study 3 (n=1,006) |
| --- | --- | --- | --- |
| Age (45+) | 0.497 | 0.499 | 0.565 |
| Woman | 0.509 | 0.507 | 0.514 |
| University | 0.322 | 0.325 | 0.273 |
| *Language* |  |  |  |
| English | 0.564 | 0.569 | 0.688 |
| French | 0.207 | 0.209 | 0.238 |
| Allophone | 0.229 | 0.222 | 0.074 |
| *Region* |  |  |  |
| Alberta | 0.118 | 0.118 | 0.112 |
| Atlantic | 0.064 | 0.065 | 0.068 |
| BC. | 0.135 | 0.137 | 0.136 |
| Prairies | 0.068 | 0.068 | 0.065 |
| Ontario | 0.381 | 0.379 | 0.384 |
| Quebec | 0.234 | 0.234 | 0.235 |

Data from three studies were weighted in order to take into account age, sex, region, language, and education. In the article, we refer to a robustness check where we do not weight the results. See Figure SM.6 for such a test.

**Tables and Figures**

Table SM2. Balance tests of randomization

|  | Study 1 (n=2,605) | | | Study 2 (n=2,350) | | | Study 3 (n=1,006) | | | |
| --- | --- | --- | --- | --- | --- | --- | --- | --- | --- | --- |
| Variables | Control | Face-saving | *t*-value | Control | Face-saving | *t*-value | Control | Face-saving | *t*-value |  |
| Age (45+) | 0.56 | 0.58 | 1.33 | 0.56 | 0.58 | -1.2 | 0.58 | 0.58 | 0.05 |  |
| Woman | 0.49 | 0.48 | 0.6 | 0.47 | 0.48 | -0.5 | 0.48 | 0.5 | -0.4 |  |
| University | 0.55 | 0.54 | 0.5 | 0.52 | 0.51 | 0.7 | 0.43 | 0.41 | 0.8 |  |
| *Language* |  |  |  |  |  |  |  |  |  |  |
| English | 0.5 | 0.53 | 1.8 | 0.54 | 0.56 | -0.8 | 0.63 | 0.68 | -1.8 |  |
| French | 0.4 | 0.39 | 0.7 | 0.38 | 0.35 | 1.3 | 0.18 | 0.17 | 0.6 |  |
| Allophone | 0.1 | 0.08 | 1.9 | 0.08 | 0.09 | -0.7 | 0.06 | 0.06 | 0.3 |  |
| *Region* |  |  |  |  |  |  |  |  |  |  |
| Alberta | 0.11 | 0.11 | -0.1 | 0.1 | 0.1 | 0.2 | 0.11 | 0.09 | 1.5 |  |
| Atlantic | 0.04 | 0.04 | 0 | 0.05 | 0.04 | 0.4 | 0.1 | 0.1 | 0.2 |  |
| BC. | 0.1 | 0.1 | 0 | 0.13 | 0.11 | **-2.1** | 0.18 | 0.12 | **2.6** |  |
| Prairies | 0.04 | 0.05 | -0.2 | 0.05 | 0.04 | 0.4 | 0.08 | 0.13 | **-2.5** |  |
| Ontario | 0.29 | 0.31 | -0.8 | 0.29 | 0.34 | **-2.1** | 0.3 | 0.38 | **-2.5** |  |
| Quebec | 0.41 | 0.4 | 0.8 | 0.4 | 0.36 | 1.8 | 0.22 | 0.18 | 1.5 |  |
| Ideology | 0.44 | 0.44 | -0.7 | 0.44 | 0.44 | -0.9 | 0.51 | 0.51 | -0.2 |  |

*Note:* t*-values were estimated from two-tailed t-test.* T*-values above 1.96 are in bold. We excluded respondents from Territories because they represented too few individuals.*

Figure SM1. The distributions of compliance, with guilty-free answer choice

*Notes: Data from Study 3.*

Figure SM2. The face-saving treatment effects, controlling for covariates

*Notes: estimations based on Table SM2. 84% confidence intervals are shown.*

Table SM3. Predicting non-compliances with covariates

|  | Study 1  ___________________________________________ | | | Study 2  ___________________________________________ | | | Study 3  ___________________________________________ | | |
| --- | --- | --- | --- | --- | --- | --- | --- | --- | --- |
|  | Visit | Home | Outdoor | Visit | Home | Outdoor | Visit | Home | Outdoor |
| Face-saving treatment | 0.14 | 0.06 | 0.30 | 0.24 | 0.09 | 0.11 | 0.92 | 0.61 | 0.69 |
|  | (0.14) | (0.13) | (0.13) | (0.12) | (0.12) | (0.11) | (0.21) | (0.19) | (0.23) |
| Older (45+ years old) | -0.55 | -0.29 | -0.28 | -0.20 | -0.15 | -0.06 | -0.54 | -0.58 | -1.03 |
|  | (0.14) | (0.13) | (0.14) | (0.12) | (0.12) | (0.11) | (0.20) | (0.19) | (0.23) |
| Sex (1=female) | -0.09 | -0.17 | 0.06 | -0.19 | -0.13 | 0.20 | -0.18 | -0.40 | -0.41 |
|  | (0.14) | (0.13) | (0.14) | (0.13) | (0.12) | (0.11) | (0.20) | (0.19) | (0.23) |
| Education (1=Uni) | 0.05 | -0.22 | 0.02 | -0.17 | -0.32 | 0.19 | 0.03 | -0.15 | -0.13 |
|  | (0.14) | (0.13) | (0.14) | (0.12) | (0.12) | (0.11) | (0.20) | (0.19) | (0.22) |
| English | 0.16 | -0.40 | -0.16 | -0.24 | -0.37 | 0.24 | 0.72 | 0.39 | 0.30 |
|  | (0.26) | (0.21) | (0.21) | (0.21) | (0.21) | (0.20) | (0.45) | (0.38) | (0.43) |
| French | 0.18 | -0.18 | -0.40 | -0.45 | -0.60 | -0.41 | 0.88 | 0.50 | 0.82 |
|  | (0.36) | (0.31) | (0.36) | (0.30) | (0.29) | (0.31) | (0.56) | (0.50) | (0.58) |
| Alberta | 0.64 | 0.66 | 0.87 | 0.15 | 0.23 | 0.49 | 0.30 | 0.84 | 0.82 |
|  | (0.34) | (0.32) | (0.37) | (0.31) | (0.31) | (0.31) | (0.51) | (0.47) | (0.59) |
| Atlantic | 0.18 | -0.12 | 0.83 | -0.46 | -0.53 | -0.33 | 0.22 | 0.29 | 0.13 |
|  | (0.43) | (0.44) | (0.43) | (0.41) | (0.41) | (0.39) | (0.48) | (0.47) | (0.62) |
| British Columbia | 0.22 | 0.20 | 1.02 | -0.21 | -0.24 | 1.04 | 0.77 | 1.00 | 1.81 |
|  | (0.37) | (0.35) | (0.37) | (0.32) | (0.31) | (0.29) | (0.45) | (0.43) | (0.50) |
| Prairies | -0.01 | 0.61 | 1.02 | -0.12 | -0.10 | 0.32 | 0.45 | 0.51 | 1.19 |
|  | (0.44) | (0.37) | (0.41) | (0.39) | (0.38) | (0.36) | (0.48) | (0.47) | (0.54) |
| Ontario | 0.00 | 0.08 | 0.94 | -0.32 | -0.31 | 0.22 | 0.48 | 0.54 | 0.75 |
|  | (0.32) | (0.30) | (0.33) | (0.28) | (0.27) | (0.27) | (0.40) | (0.39) | (0.47) |
| Left-right ideology | 0.43 | 0.08 | 0.22 | 0.52 | -0.01 | 0.19 | 0.43 | 0.44 | 0.98 |
|  | (0.32) | (0.30) | (0.32) | (0.29) | (0.28) | (0.26) | (0.44) | (0.41) | (0.49) |
| Constant | -2.49 | -1.70 | -2.80 | -1.50 | -0.97 | -2.12 | -3.28 | -2.55 | -3.41 |
|  | (0.41) | (0.37) | (0.41) | (0.35) | (0.34) | (0.34) | (0.65) | (0.59) | (0.70) |
| Observations | 2590 | 2591 | 2592 | 2344 | 2343 | 2345 | 901 | 901 | 901 |
| Pseudo *R*^2^ | 0.018 | 0.014 | 0.042 | 0.013 | 0.013 | 0.052 | 0.048 | 0.043 | 0.088 |

*Notes: Models are logistic regressions predicting Visit (Visit someone else’s home), Home (Have someone over at your home who does not live with you) and Outdoor (Get together outdoor with people who do not live with you). Coefficients are standard errors are shown. The reference category for the language is ‘other’ and the reference category for the region is ‘Quebec’.*

Figure SM3. Moderation effects in Study 1

*Note: 84% confidence intervals are shown.*

Figure SM4. Moderation effects in Study 2

*Note: 84% confidence intervals are shown.*

Figure SM5. Moderation effects in Study 3

*Note: 84% confidence intervals are shown.*

Figure SM6. Replication of Figure 1 without weight

*Note: 84% confidence intervals are shown.*

Figure SM7. Other items included in all studies

*Note: 84% confidence intervals are shown.*

Figure SM8. The face-saving treatment effect across languages

*Note: 84% confidence intervals are shown.*
